# WAPL orchestrates porcine oocyte meiotic progression via control of spindle assembly checkpoint activity

**DOI:** 10.1186/s12958-021-00740-1

**Published:** 2021-04-19

**Authors:** Changyin Zhou, Yilong Miao, Xue Zhang, Bo Xiong

**Affiliations:** College of Animal Science and Technology, Nanjing Agricultural University, 210095 Nanjing, China

**Keywords:** Cohesin, WAPL, SAC, BUB3, Porcine oocytes

## Abstract

**Background:**

In mitotic cells, WAPL acts as a cohesin release factor to remove cohesin complexes from chromosome arms during prophase to allow the accurate chromosome segregation in anaphase. However, we have recently documented that Wapl exerts a unique meiotic function in the spindle assembly checkpoint (SAC) control through maintaining Bub3 stability during mouse oocyte meiosis I. Whether this noncanonical function is conserved among species is still unknown.

**Methods:**

We applied RNAi-based gene silencing approach to deplete WAPL in porcine oocytes, validating the conserved roles of WAPL in the regulation of SAC activity during mammalian oocyte maturation. We also employed immunostaining, immunoblotting and image quantification analyses to test the WAPL depletion on the meiotic progression, spindle assembly, chromosome alignment and dynamics of SAC protein in porcine oocytes.

**Results:**

We showed that depletion of WAPL resulted in the accelerated meiotic progression by displaying the precocious polar body extrusion and compromised spindle assembly and chromosome alignment. Notably, we observed that the protein level of BUB3 was substantially reduced in WAPL-depleted oocytes, especially at kinetochores.

**Conclusions:**

Collectively, our data demonstrate that WAPL participates in the porcine oocyte meiotic progression through maintenance of BUB3 protein levels and SAC activity. This meiotic function of WAPL in oocytes is highly conserved between pigs and mice.

## Background

Oocyte meiosis is a special type of cell division, which includes a single round of DNA replication, followed by two successive rounds of chromosome segregation (meiosis I and meiosis II) [1, 2]. The extrusion of first polar body indicates the completion of oocyte meiosis I, and fusion of sperm with the matured oocytes triggers the completion of meiosis II [3, 4]. During meiotic progression, the spindle assembly checkpoint (SAC), a surveillance system that monitors the attachment of spindle microtubules to kinetochores, prevents inaccurate chromosome segregation to ensure formation of normal and competent oocytes [5–9]. Compromised SAC activity accelerates the oocyte meiotic progression and thus results in the aneuploid eggs [5, 6].

Chromosome cohesion is another mechanism that is required for faithful chromosome segregation in cells. This biological event is mediated by a ring-shaped complex cohesin which consists of four core components: SMC1, SMC3, SCC1 and SCC3 [10–12]. Besides sister chromatid cohesion and chromosome segregation, cohesin and its regulatory factors also exert functions in a variety of other biological processes such as DNA damage repair, transcriptional regulation and chromatin architecture organization [11–15]. Chromosome cohesion involves multiple steps including cohesin loading, cohesion establishment, cohesion maintenance and cohesion dissolution [11]. In late G1 phase, cohesin loads onto chromosomes with the help of the evolutionarily conserved loading factor NIPBL/MAU2 [11]. By S phase, cohesin is acetylated on SMC3 subunit by the acetyltransferases ESCO1 and ESCO2 to establish the cohesion [16]. Then, acetylated SMC3 recruits Sororin to PDS5 to antagonize WAPL (wings apart-like protein) function and stabilize cohesin on chromosomes [17]. After G2/M transition, binding of WAPL to PDS5 displaces phosphorylated Sororin and remove a mass of cohesin complexes from chromosome arms [18]. Lastly in anaphase, Separase cleaves cohesin SCC1 subunit to allow the cohesion dissolution [19]. Therefore, the removal of cohesin complexes from chromosome arms by the cohesin release factor WAPL during prophase is a prerequisite for chromosome segregation [18, 20, 21]. Loss of WAPL delays the early stages of cell cycle and impairs the mitotic progression [20, 21]. Furthermore, homozygous knockout of Wapl exhibits embryonic lethality in mice, suggesting that Wapl is indispensable for the mammalian embryonic development [22]. Interestingly, we have previously discovered a noncanonical role of Wapl in mediating SAC activity through maintenance of Bub3 stability during mouse oocyte meiosis I [23]. Here, we employed porcine oocytes as a model to investigate whether the unique function of WAPL beyond chromosome cohesion in oocyte meiosis is conserved among species.

## Materials and methods

### Antibodies

Rabbit polyclonal anti-WAPL antibody was purchased from Proteintech Group (Rosemont, IL, USA; Cat# 16370-1-AP); rabbit monoclonal anti-BUB3 antibody was purchased from Abcam (Cambridge, MA, USA; Cat# ab133699); human anti-centromere antibody was purchased from Antibodies Incorporated (Davis, CA, USA; Cat# CA95617); mouse monoclonal anti-α-tubulin-FITC antibody were purchased from Sigma (St. Louis, MO, USA; Cat# F2168); rabbit monoclonal anti-GAPDH antibody was purchased from Cell Signaling Technology (Danvers, MA, USA; Cat# 2118).

### Collection of porcine oocytes

Porcine ovaries were obtained from a local abattoir and transported to the laboratory in a physiological saline containing streptomycin sulphate and penicillin G within 2 h after slaughtering. Cumulus-oocyte complexes (COCs) were aspirated from the follicles using a disposable syringe. COCs with a compact cumulus cells were selected for *in vitro* maturation (IVM). The maturation medium is TCM-199 (ThermoFisher Scientific, Waltham, MA, USA; Cat# 11,150,059) supplemented with 10 % porcine follicular fluid, 5 µg/mL insulin, 10 ng/mL EGF, 0.6 mM cysteine, 0.2 mM pyruvate, 25 µg/mL kanamycin and 10 IU/mL of each eCG and hCG. 20 GV COCs were cultured in a drop of 100 µL maturation medium covered with mineral oil at 38.5 °C, 5 % CO2 for 26–28 h to metaphase I stage and for 42–44 h to metaphase II stage.

### WAPL knockdown

Knockdown of WAPL in porcine oocytes was achieved via microinjection of 50 µM porcine WAPL-targeting siRNA (Genepharma, Shanghai, China). WAPL siRNA antisense sequence is UUCUUUGCCUGAUUCAGGCTT. Twenty oocytes per group were transferred to 30 µl droplets of manipulation medium (TCM-199 supplemented with 0.6 mM NaHCO3, 10 mM HEPES, 30 mM NaCl, and 0.1 % BSA). The oocytes were held in place using a holding pipette, and the plasma membrane was penetrated by the injection pipette via which a constant medium flow of siRNA was injected until swelling was obvious.

### Immunofluorescent and confocal microscopy

DOs were incubated in the fixation solution (4 % paraformaldehyde/PBS) for 30 min, in the permeabilization solution (1 % Triton X-100/PBS) for 1 h, and in the blocking solution (1 % BSA-supplemented PBS) for 1 h at room temperature (RT), followed by incubation with anti-WAPL antibody (1:100), anti-BUB3 antibody (1:100), anti-centromere antibody (1:200) and α-tubulin-FITC antibody (1:200) overnight at 4 °C. After washes in PBST, oocytes were incubated with the corresponding secondary antibodies for 1 h and counterstained with 10 µg/ml Hoechst 33,342 or propidium iodide (PI) for 10 min at RT. Lastly, oocytes were mounted on the glass slides and imaged under a confocal microscope (LSM 700 META, Zeiss, Germany).

### Quantification of spindle area and chromosome width

ImageJ software (NIH, Bethesda, MD, USA) was applied to measure the spindle area by circling the entire spindle apparatus, and measure the metaphase I plate width by quantifying the maximal distance between the two lines that were drawn parallelly to the metaphase I plate and tangentially to the extreme limits of the chromosomes.

### Immunoblotting analysis

Porcine oocytes were collected in the lysis buffer (4 × LDS sample buffer, ThermoFisher Scientific; Cat# NP0007) with protease inhibitor and heated at 95 °C for 5 min. Proteins were separated on 10 % precast gels (Bis-Tris) and transferred to PVDF membranes. The blots were then incubated in the blocking buffer (5 % low fat dry milk/TBST) for 1 h at RT and then probed with anti-WAPL antibody (1:1000), anti-BUB3 antibody (1:1000), anti-α-tubulin antibody (1:1000) or anti-GAPDH antibody (1:5000) overnight at 4 °C. After washes in TBST, the blots were incubated with the corresponding secondary antibodies for 1 h at RT. Chemiluminescence signals were acquired with ECL Plus (ThermoFisher Scientific; Cat# 32,132) and protein bands were detected by Tanon-3900 Imaging System.

### Statistical analysis

All percentages or values from at least three repeated experiments were expressed as mean ± SEM or mean ± SD, and the number of oocytes observed was labeled in parentheses as (n). Data were analyzed by paired samples t-test, which was provided by GraphPad Prism 5 statistical software. The level of significance was accepted as *P* < 0.05.

## Results

### Localization and expression patterns of WAPL in porcine oocytes

**Fig. 1:**
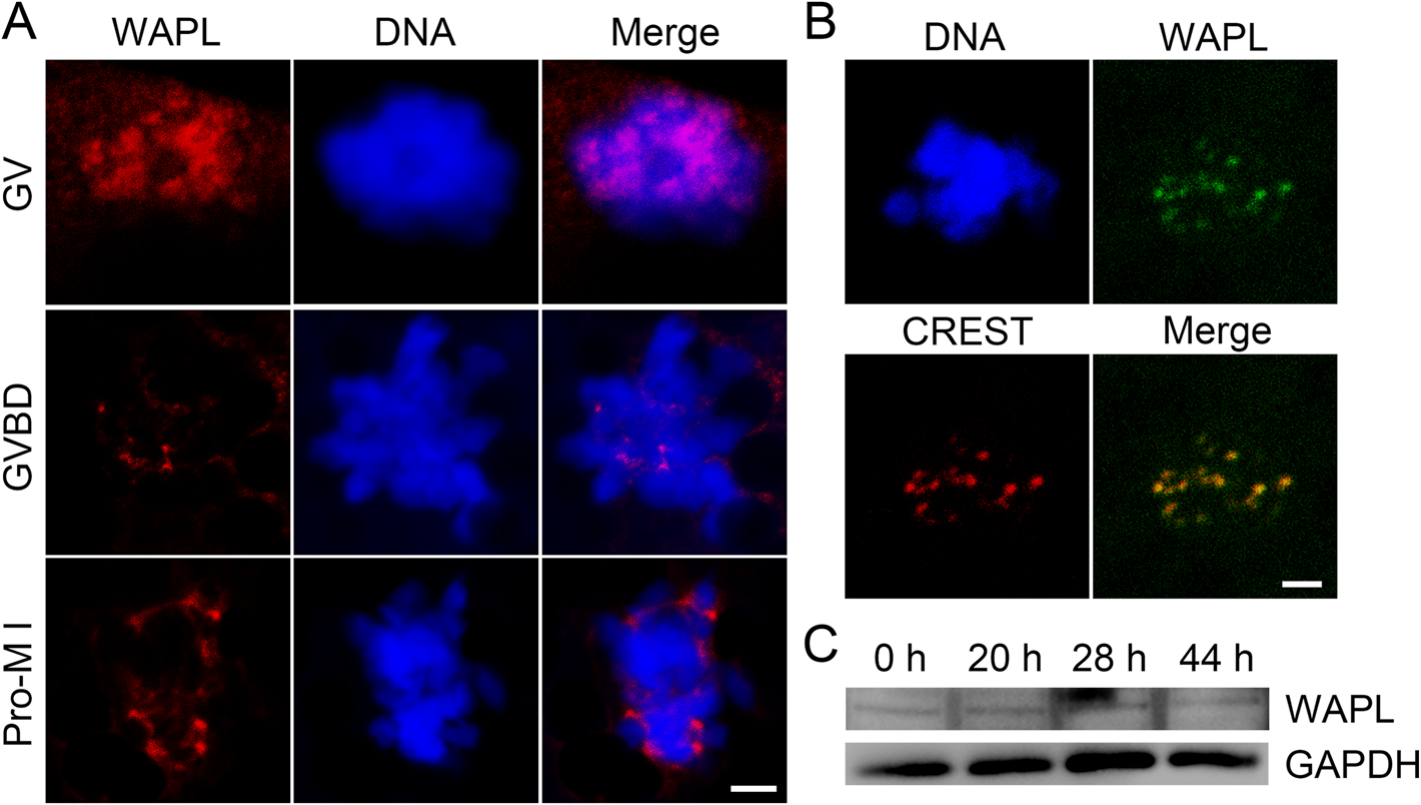
Subcellular localization and protein expression of WAPL during porcine meiotic maturation. **a** Representative localization of endogenous WAPL in porcine oocytes. Porcine oocytes at different developmental stages were immunostained with WAPL antibody and counterstained with Hoechst. Scale bar, 2.5 μm. **b** Representative image of co-localization of WAPL with kinetochores in porcine oocytes. Porcine oocytes at pro-metaphase I (Pro-M I) stage were immunostained with WAPL and CREST antibodies and counterstained with Hoechst. Scale bar, 2 μm. **c** Protein levels of WAPL during porcine oocyte meiotic maturation corresponding to GV, GVBD, metaphase I (M I) and metaphase II (M II) stages were examined by immunoblotting. The blots were probed with WAPL and GAPDH antibodies, respectively

We firstly examined the subcellular localization and protein expression patterns of WAPL to predict its specific roles during porcine oocyte meiosis. The immunofluorescent analysis by antibody staining showed that endogenous WAPL mainly accumulated in the germinal vesicle (GV) of GV oocytes (Fig. 1 a). Upon germinal vesicle breakdown (GVBD), WAPL distributed on the chromosome and specifically concentrated at kinetochores (Fig. 1 a, b) at prometaphase I (pro-M I) stage. The protein expression level as assessed by immunoblotting analysis revealed that the WAPL amount remained constant at different developmental stages during porcine oocyte meiotic maturation (Fig. 1 c).

### Depletion of WAPL accelerates the meiotic progression during porcine oocyte meiosis

**Fig. 2:**
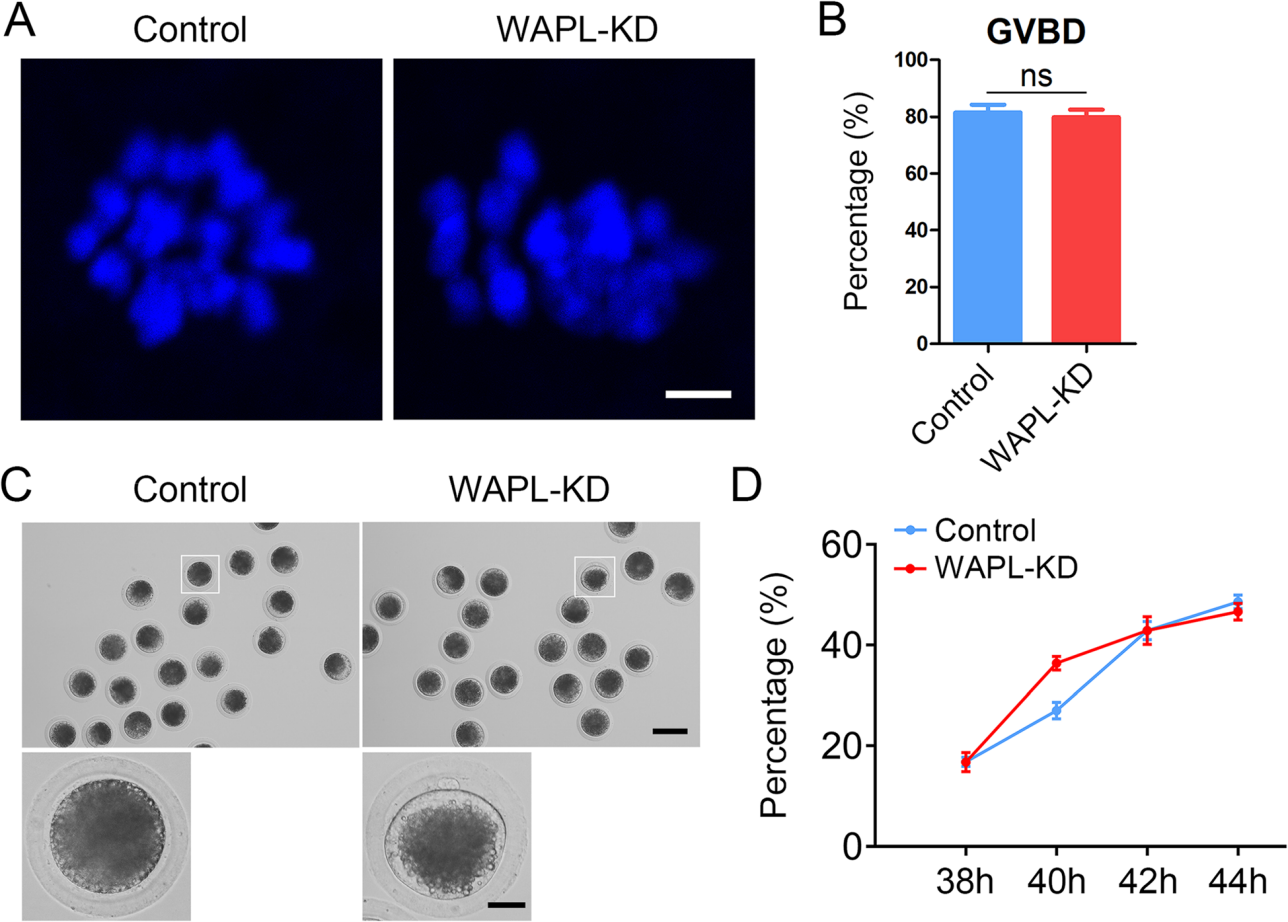
Effect of WAPL depletion on the meiotic progression of porcine oocytes. **a** Representative images of chromosomes in oocytes after GVBD in control and WAPL-KD groups. Scale bar, 2.5 μm. **b** The incidence of GVBD was quantified in control (*n* = 82) and WAPL-KD (*n* = 85) oocytes. **c** Representative images of oocytes with first polar body at time point of 40 h following *in vitro* culture were shown in control and WAPL-KD groups. Scale bars, 100 μm, 25 μm. **d** Quantitative analysis of polar body extrusion rate was shown in control (*n* = 107) and WAPL-KD (*n* = 107) oocytes at consecutive time points from 38 to 44 h during *in vitro* maturation

To evaluate the effect of WAPL depletion on the meiotic progression of porcine oocytes, we recorded the frequency of GVBD and polar body extrusion (PBE), two key developmental events indicating the resumption and completion of meiosis I. We observed that depletion of WAPL did not disturb the resumption of meiosis by showing the comparable rate of GVBD between WAPL-depleted oocytes and controls (Fig. 2 a, b). After *in vitro* maturation for 44 h, the rate of PBE was also not affected in WAPL-depleted oocytes (Fig. 2 c, d). However, a significantly higher incidence of PBE was found in WAPL-depleted oocytes than that in controls at 40 h following *in vitro* culture from GV stage (Fig. 2 c, d), implying that the meiotic progression was accelerated and SAC was inactivated in the porcine oocytes depleted of WAPL.

### Depletion of WAPL results in reduced protein level of BUB3 in porcine oocytes

**Fig. 3:**
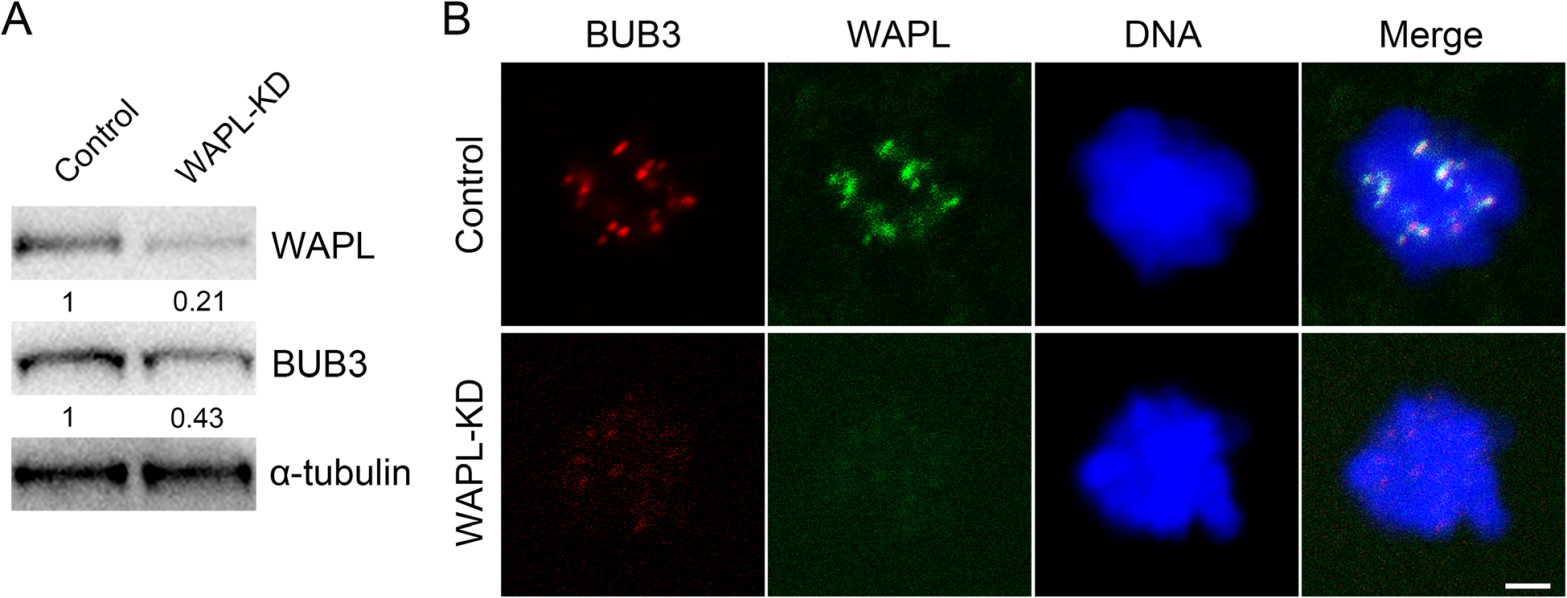
Effect of WAPL depletion on the protein level and localization of BUB3 in porcine oocytes. **a** Immunoblotting analysis of BUB3 in control and WAPL-KD oocytes. 120 oocytes were collected and immunoblotted for BUB3, WAPL and α-tubulin. The band intensity of WAPL and BUB3 was normalized with α-tubulin. **b** Representative images of BUB3 localization in control or WAPL-KD oocytes. Porcine oocytes at Pro-M I stage were immunostained for BUB3 and WAPL, and counterstained for DNA. Scale bar, 2.5 μm

Our previous study has reported that the role of WAPL in regulation of SAC activity in mouse oocytes is mediated by a pivotal component of SAC Bub3 [23]. To ask whether this mechanism is conserved in porcine oocytes, we assessed the expression and localization of BUB3 after WAPL depletion. The immunoblotting data validated that RNAi-based gene silencing effectively suppressed the protein expression of WAPL in porcine oocytes (Fig. 3 a). Particularly, the protein level of BUB3 was prominently reduced in WAPL-depleted oocytes compared to the controls (Fig. 3 a). In addition, the immunostaining result further revealed that BUB3 at kinetochores almost completely disappeared in WAPL-depleted oocytes (Fig. 3b), suggesting that WAPL is required for the maintenance of BUB3 abundance in porcine oocytes.

### WAPL is essential for proper spindle assembly and chromosome alignment in porcine oocytes

**Fig. 4:**
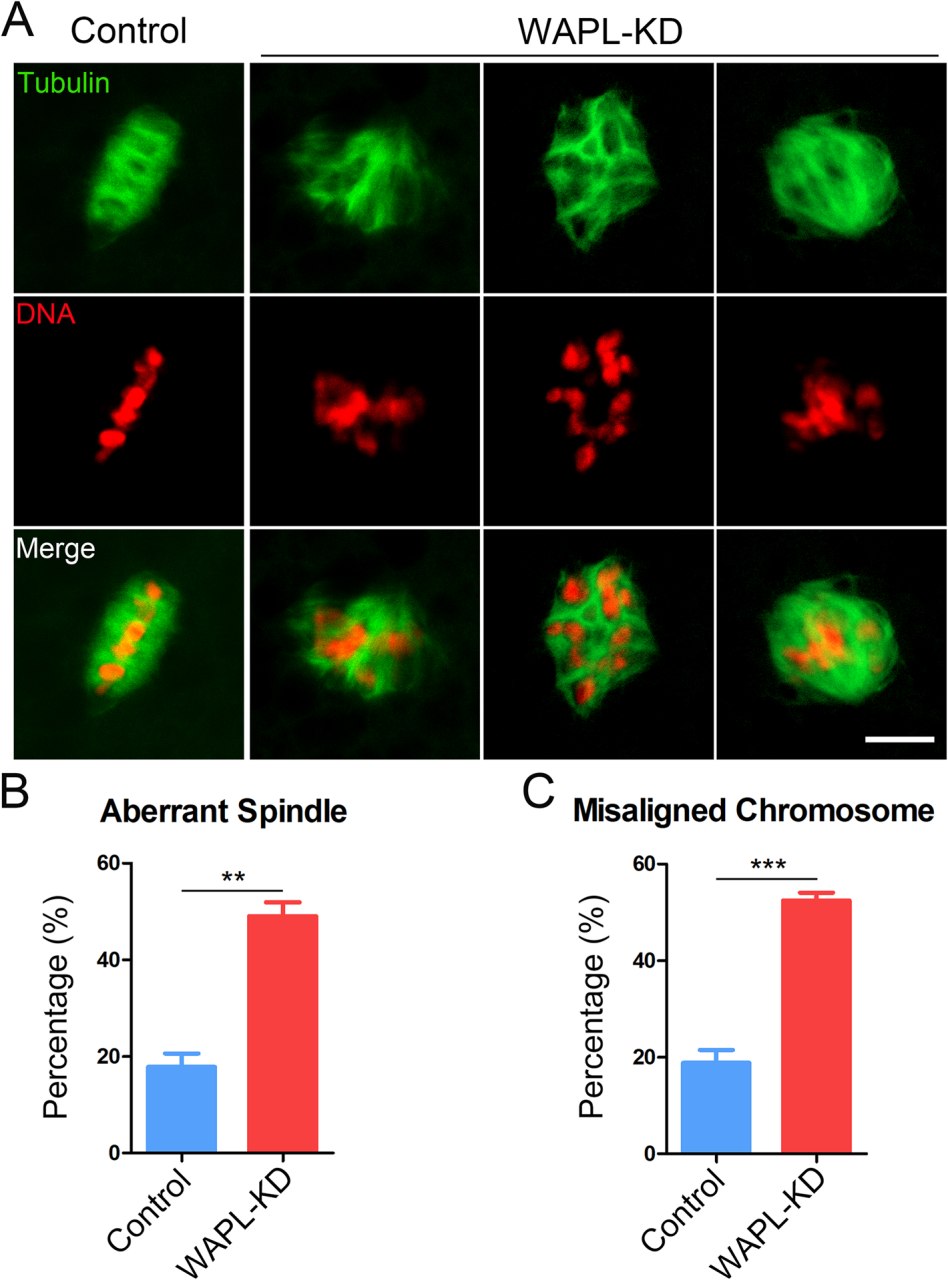
Effect of WAPL depletion on the spindle/chromosome structure in porcine oocytes. **a** Representative images of spindle morphologies and chromosome alignment in control and WAPL-KD oocytes. Scale bar, 5 μm. **b** The proportion of defective spindles was recorded in control (*n* = 73) and WAPL-KD (*n* = 82) oocytes. **c** The proportion of misaligned chromosomes was recorded in control (*n* = 73) and WAPL-KD (*n* = 82) oocytes. Data of (**b**) and (**c**) were presented as mean percentage (mean ± SEM) of at least three independent experiments. ** *P* < 0.01, *** *P* < 0.001

As impairment of SAC activity usually leads to the defects of spindle assembly, we next tested whether this is the case in WAPL-depleted porcine oocytes. To this end, Oocytes at M I stage from control and WAPL-depleted groups were immunostained with anti-α-tubulin antibody to show the spindle structure and counterstained with PI to display the chromosome alignment. As shown in Fig. 4 a, a considerably increased frequency of spindle disorganization and chromosome misalignment was observed in WAPL-depleted oocytes, exhibiting the diverse malformed spindle morphologies with several scattered or lagging chromosomes (Fig. 4 a-c). On the contrary, control oocytes showed a normally organized spindle apparatus with well-aligned chromosomes at the equatorial plate (Fig. 4 a).

**Fig. 5:**
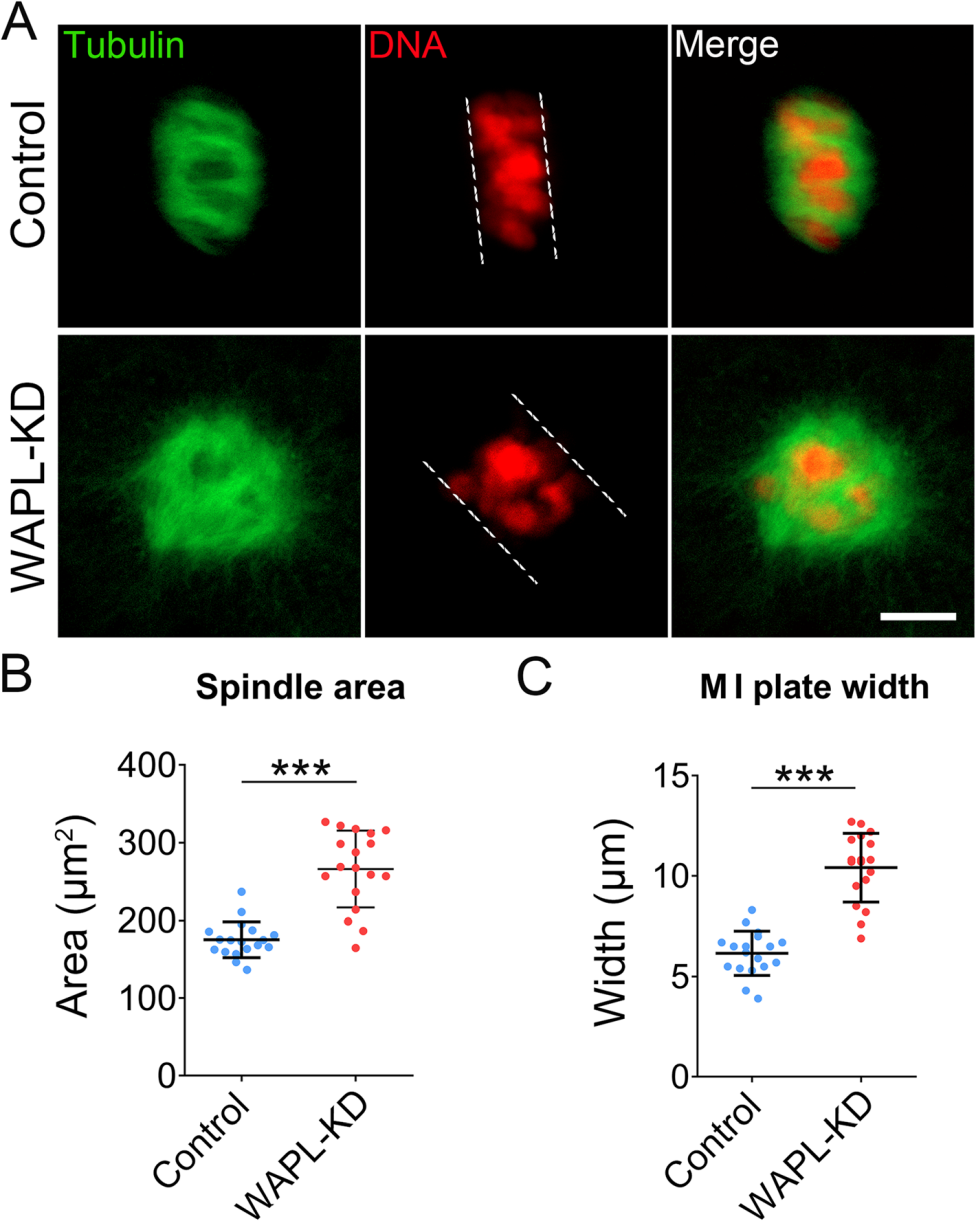
Effect of WAPL depletion on the spindle area and M I plate width in porcine oocytes. **a** Representative images of spindle area and chromosome width in control and WAPL-KD oocytes. Scale bar, 5 μm. **b** The spindle area was measured in control (*n* = 18) and WAPL-KD (*n* = 18) oocytes. **c** The width of M I plate was measured in control (*n* = 18) and WAPL-KD (*n* = 18) oocytes. Data of (**b**) and (**c**) were presented as mean percentage (mean ± SD) of at least three independent experiments. *** *P* < 0.001

We further quantified the spindle area and M I plate width of porcine oocytes to reflect the status of spindle assembly and chromosome alignment. We found that the spindle area was substantially larger in WAPL-depleted oocytes than that in control oocytes (Fig. 5 a, b). Moreover, the distance of the chromosome plate at M I stage also became considerably wider in WAPL-depleted oocytes compared to the controls (Fig. 5 a, c), indicating that spindle/chromosome structure was compromised by WAPL depletion in porcine oocytes.

## Discussion

In mitotic cells, most cohesin complexes on the chromosome arms are removed by WAPL [18, 20, 24, 25] during prophase and prometaphase to ensure accurate chromosome segregation in anaphase. However, interestingly, in mammalian oocytes, homologous chromosomes become paired to form a bivalent in meiotic prophase [6, 26], and subsequently the distal cohesins on bivalent arms are stripped by Separase instead of Wapl to allow homologous chromosome segregation at anaphase I stage [6, 27]. In our recent study, we have unexpectedly found that Wapl functions as a SAC regulator by maintaining the stability of Bub3 to orchestrate the mouse oocyte meiotic progression [23]. Nevertheless, whether this noncanonal role of Wapl in oocyte meiosis is conserved among species still needs to be investigated.

To determine it, we initially examined the subcellular localization and protein expression of WAPL during porcine oocyte maturation. Consistent with the localization pattern in mouse oocytes [23], WAPL distributed in the nucleus of GV oocytes and then translocated to chromosomes with accumulation at kinetochores after GVBD. However, the protein expression trend of WAPL during porcine oocyte meiosis is not totally the same as those in mice [23]. WAPL levels remain constant throughout the meiotic maturation in porcine oocytes but exhibit increasingly upregulated in mouse oocytes. This difference might be due to the distinct protein metabolism of WAPL between porcine and mouse oocytes. It is also possible that there are additional functions of WAPL in mouse or porcine oocytes that we have not identified.

The similar localization patterns of WAPL in oocytes from two species indicate that it may have conserved functions during female meiosis. To validate this possibility, we employed gene-targeted siRNA microinjection to specifically knock down WAPL in porcine oocytes. As anticipated, we found that depletion of WAPL did not markedly affect GVBD and first polar body extrusion of porcine oocytes, but accelerated the meiotic progression, indicative of impaired SAC activity. In addition, our findings revealed that the protein abundance of BUB3 was substantially reduced in WAPL-depleted porcine oocytes, especially at kinetochores, which photocopies the mechanism underlying the Wapl-mediated SAC control in mouse oocytes. Accordingly, depletion of WAPL resulted in the defects of spindle assembly and chromosome alignment during porcine oocyte maturation. Therefore, these observations suggest that the unique functions of WAPL during oocyte meiosis are highly conserved between pigs and mice.

## Conclusions

Collectively, our findings not only reveal the biological significance of WAPL in livestock reproduction but also extend the understanding of the potential etiology of human oocyte maturation defects, because porcine oocytes are developmentally and physiologically more close to human oocytes than mouse ones.

## Data Availability

All data generated or analyzed during this study are available from the corresponding author on reasonable request.

## Abbreviations

SAC: Spindle assembly checkpoint
GV: Germinal vesicle
GVBD: Germinal vesicle breakdown
Pro-M I: Prometaphase I
PBE: Polar body extrusion
IVM: In vitro maturation
COCs: Cumulus-oocyte complexes
DOs: Denuded oocytes
PI: Propidium iodide
RT: Room temperature
